# Everybody's Page

**Published:** 1888-01-14

**Authors:** 


### Everybody's Page.

If all be true that is said of it, the kola nut is one of the most wonderful and beneficent products of Nature. Here is a list of its various properties. It is a food more nutritious than cocoa, and when mixed with cocoa paste, makes a chocolate of exceptionally fine flavour. It preserves nerve energy to such an extent that after a single cup of kola chocolate a labourer can go through his day's work without any sense of fatigue. This quality, with its consequent economy of tissue, makes the kola invaluable in wasting diseases. It also exercises a purifying effect on foul water, and may therefore be regarded as a preventive of disease. It clarifies beer and spirits, and, it is said, removes the objectionable flavour from tainted meat. As if this were not enough, it has the remarkable effect of curing the effect on head and stomach of the deep potations which unfortunately characterise our nation. Let not the drunkard, however, think that he can indulge in his favourite vice without consequence of any sort. The morning headache that succeeds the night's debauch is indeed removed by the aid of kola, but in its place has come a peculiar aversion from strong drink. Truly, this kola is a wonder-working drug. The narrative of its powers reads like one of the accounts of universal specifics that glorify by their imaginative power the dull pages of our newspapers; yet each quality has been proved by scientific experiment. Kola has long been used in the interior of Africa in the preparation of a beverage and as a sacred symbol—the white kola being a token of peace, the red implying war; but now that its various uses are becoming known it will doubtless be extensively cultivated in all our tropical colonies.

---

One of the most disagreeable characteristics of many skin diseases is the fact that the patient is not permitted to use soap, because the free alkali in it has an irritating effect which militates against recovery. To overcome this discomfort Dr. Unna, of Hamburg, has introduced into medical practice a soap which contains three or four per cent. of oil more than is needed for combining with the potash to form soap. This "over-fatty soap," as the inventor terms it, may be used in skin diseases where ordinary soap cannot be employed. It may also be advantageously used by all persons with delicate skins during the winter months, as the superabundance of oil remains as a thin, invisible layer on the skin, and effectually prevents chapping or roughness of any kind.

---

The power of mind over body has lately been very sadly proved at Southport, Lancashire, where a little girl has died from congestion of the brain, brought on by terror. The child was told that a ghost which had been seen at Liverpool would shortly appear at Southport, and the fear of the apparition is alleged to have brought on the illness which killed her. A case like this is sufficiently startling to frighten people for a time into more consideration for the timid and credulous imaginations of children than they often display; but the public memory is short, and probably before long nursemaids and even parents will recur to the habit of frightening children into silence and obedience by threats of ghosts and bogeys of all sorts. Even when the consequences of such cruel cheats are not immediately apparent, they are more far-reaching than could be guessed. We are ourselves acquainted with a gentleman, no coward by daylight, who cannot overcome a superstitious terror of darkness implanted thirty years ago by an unscrupulous nurse.

---

It is not generally known that the reason hair curls when wound on a hot iron is that the moisture on the side next the iron being evaporated by the heat, the cells in that part approach each other more closely, and this shrinking of one side forms a bend or curve.

---

The November number of the "Proceedings of the Society for the Study of Inebriety" consists of an interesting paper on "The Effects of Moderate Drinking on the Human Constitution," by Dr. George Harley, F.R.S. After explaining the difficulty of collecting statistics on the subject of so-called "moderate drinking," Dr. Harley gave as an approximate test the death-rate among persons directly connected with the liquor trade as compared with that of those whose work does not lay them open to such a temptation. Quoting from the Registrar-General's report on the subject he said: "The comparative mortality figure for brewers is 1,361; for innkeepers, publicans, and dealers in wines, spirits, or beers, 1,521; and for inn and hotel servants, waiters, etc., no less than 2,205; whereas for maltsters, who are only concerned with the materials and not with the liquor itself, the figure is only 830." Dr. Harley considers alcohol a narcotic poison, the primary exciting effects being similar to those produced by opium, though longer in duration. The direct action of alcohol as a poison is that of interfering with the due oxidation of the blood, and hindering the functions of the liver. The liver being rendered incapable of doing its work, bile is generated; and there is scarcely a more formidable cerebral poison than bile. The pleasant feelings induced at first by the use of alcohol are due, Dr. Harley thinks, to the comparative loss of nerve sensibility, increasing, with increased indulgence, to utter unconsciousness. It may be thought from this that Dr. Harley objects strongly to alcohol in any form; but this is not the case. He admits the value of it in many diseases, especially in cardiac syncope, where it not only stimulates the heart's action, but increases the flow of blood through the vessels of the brain. Moreover, while denying that pure alcohol can be regarded as a food, he admits the nutritive value of wines and malt liquor. Therefore he is not to be regarded as an advocate of total abstinence; though, in face of his statistics, those who read his pamphlet will feel much inclined to follow his advice and make their "moderate drinking" very moderate indeed.

---

Will women ever understand questions of finance, or—which would be almost equally good—learn that they don't know anything about them! A story, recently published in the *Pall Mall Gazette*, and vouched for by a county coroner, points a sad moral. A young widow, being in want of £20, borrowed it from a money-lender. He would not give less than £30, as the security was to be a bill of sale on her furniture, which would not be valid for a less sum. She was also to pay interest at the rate of sixty per cent. per annum! The poor woman agreed to these terms, paid back £10 within a day or two after receiving the money, and went on paying 10*s.* weekly till October 4th. To do this, however, she had to run into debt for necessaries, and in October, her resources and her credit being alike exhausted, she committed suicide by hanging herself, leaving five children. As she was possessed of a small freehold, she could easily have raised the money by mortgage at five per cent. interest; but this does not seem to have occurred to her. Yet surely she must have had some friend who would have given her good and disinterested advice. In spite of all that cynics say, the world is not barren of good and honest men; but it is not wise to seek for them among usurers. It will be a good day for women, and it is not far distant we hope, when they are taught the practical business of life in every department in which they are likely to be called upon to play an active part.

